# Traffic-related air pollution is a risk factor in the development of chronic obstructive pulmonary disease

**DOI:** 10.3389/fpubh.2022.1036192

**Published:** 2022-12-07

**Authors:** Jinzhen Zheng, Sha Liu, Jieqi Peng, Huanhuan Peng, Zihui Wang, Zhishan Deng, Chenglong Li, Naijian Li, Longhui Tang, Jianwu Xu, Jingwen Li, Bing Li, Yumin Zhou, Pixin Ran

**Affiliations:** ^1^State Key Laboratory of Respiratory Disease, National Clinical Research Center for Respiratory Diseases, The Guangzhou Institute of Respiratory Health, and The First Affiliated Hospital of Guangzhou Medical University, Guangdong, China; ^2^Department of Pulmonary and Critical Care Medicine, Shantou Central Hospital, Shantou, Guangdong, China; ^3^The Second Affiliated Hospital, Hengyang Medical School, University of South China, Hengyang, Hunan, China; ^4^Guangzhou Laboratory, Bio Island, Guangzhou, Guangdong, China; ^5^Department of Pulmonary and Critical Care Medicine, The Fourth People's Hospital of Chenzhou, Chenzhou, Hunan, China; ^6^Department of Pulmonary and Critical Care Medicine, Chenzhou Third People's Hospital, Chenzhou, Hunan, China; ^7^College of Life Sciences, Guangzhou Medical University, Guangzhou, Guangdong, China

**Keywords:** chronic obstructive pulmonary disease (COPD), air pollution, traffic pollution, risk factors, health

## Abstract

**Background:**

Outdoor traffic-related air pollution has negative effects on respiratory health. In this study, we aimed to explore the effect of outdoor traffic-related air pollution on chronic obstructive pulmonary disease (COPD) in Guangzhou.

**Methods:**

We enrolled 1,460 residents aged 40 years or older between 21 January 2014 and 31 January 2018. We administered questionnaires and spirometry tests. The distance of participants' residences or locations of outdoor activities from busy roads (as indicators of outdoor traffic-related air pollution), indoor air pollution, and smoking history were queried in the questionnaires.

**Results:**

Of the 1,460 residents with valid survey and test results, 292 were diagnosed with COPD, with a detection rate of 20%. Participants who lived and did their outdoor activities near busy roads had a higher detection rate of COPD. Among residents living at distances of <50 meters, 50–199 meters, and more than 200 meters from busy roads, the detection rates were 20.6, 21.2, and 14.8%, respectively; the rates for outdoor activities at these distances were 23.8, 24.5, and 13.7%, respectively (*p* < 0.05). After adjusting for sex, age, smoking status, family history, and smoking index, the distance of outdoor activities from busy roads was an independent risk factor for COPD. Participants whose outdoor activities were conducted <50 meters and 50–199 meters of main roads had odds ratios of 1.54 (95% confidence interval 1.01–2.36) and 1.84 (95% interval 1.23–2.76) for the risk of COPD in comparison with a distance of more than 200 meters from busy roads.

**Conclusions:**

Residents of Guangzhou whose outdoor activities were close to busy roads had a high risk of COPD. Traffic-related air pollution presents a risk to human health and a risk of COPD.

## Introduction

Chronic obstructive pulmonary disease (COPD) is the fourth leading cause of death worldwide, characterized by persistent respiratory symptoms and irreversible airflow (1). COPD usually involves airway or alveolar abnormalities caused by noxious particles or exposure to gases (2). Exposure to noxious particles or gases (e.g., *via* smoking, household wood burners, fires, or environmental pollutants) is the main risk factor for COPD (3). The European Study of Cohorts for Air Pollution Effects project showed that greater exposure to ambient nitrogen dioxide and exposure to particulate matter (PM) with a 50% cutoff aerodynamic diameter of 10 μm (PM_10_) were related to lower levels of forced expiratory volume in 1 sec (FEV1) and forced vital capacity (FVC) (4). Traffic-related air pollution is an important source of atmospheric pollution, especially PM, sulfur dioxide, nitrogen oxides, and carbon monoxide (5). It has been reported that outdoor traffic-related air pollution negatively affects respiratory health in Europe and the United States, where pollution is mild (6). It is, therefore, crucial to reducing the emission of air pollution.

Air pollution could increase the risk of respiratory mortality, with a negative association between lung function and obstructive lung diseases. It has been reported that incomplete combustion of biomass fuel could result in COPD (7). Each 10-μg/m3 increase in fine PM is linked to a 3.1% increased risk of hospitalization and a 2.5% increase in mortality (8). In China, studies on the association of traffic-related air pollution with risk factors for COPD remain scarce, especially in community districts. We aimed to explore whether traffic-related air pollution (assessed according to the distance from the main road) affects lung function and whether it is a risk factor for developing COPD in a population of community residents of Guangzhou.

## Materials and methods

### Study design and participants

**Figure 1:**
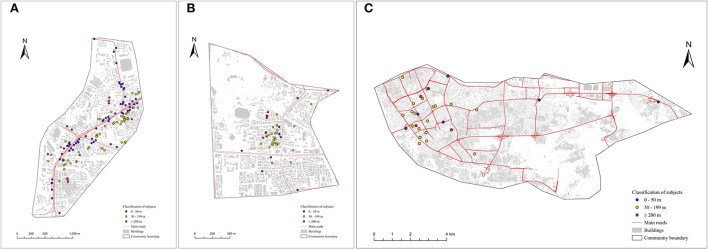
**(A–C)** Classification of subjects based on distance of each home from the main road in Nanyuan, Hongqiao and Xingang Community. The three maps represented classification of subjects according to distance of each home from the main road (<50 m, 50–199 m, and ≥200 m groups) in Nanyuan, Hongqiao and Xingang Community.

This was a cross-sectional observational analysis of a prospective study. We requested that doctors working in community hospitals ask local residents to participate in our project. Between 21 January 21 2014 and 31 January 31 2018, we recruited permanent residents in three communities in Guangzhou's central areas (Figure 1), including the Nanyuan community in Liwan District, the Hongqiao community in Yuexiu District, and the Xingang community in Haizhu District. We included participants aged 40–80 years who could complete the questionnaires and lung function tests. We excluded participants who had undergone thoracic, abdominal, or eye surgery or experienced myocardial infarction in the past 3 months; those who had been hospitalized for COPD exacerbation in the past 4 weeks; those who had received antibacterial treatment or chemotherapy for active tuberculosis or a newly discovered tumor; and those with other respiratory diseases, except for asthma, such as lung cancer, pneumoconiosis, and extensive bronchiectasis.

### Questionnaire

The questionnaire related to air pollution was revised from the National German Health Survey (9) and the Air Pollution on Lung, Inflammation, and Aging (SALIA) study (10) based on a standardized questionnaire from the international BOLD (Burden of Obstructive Lung Diseases) study. The study questionnaire included demographic characteristics (age, sex, and body mass index [BMI]), tobacco smoking history, family history of respiratory disease, the distance of residence or location of outdoor activities from busy roads, medical history, history of medical therapy, chronic respiratory symptoms, and frequency of acute COPD exacerbations during the preceding year. The smoking index was calculated as smoking years multiplied by the number of packs of cigarettes smoked (pack-years). A family history of respiratory diseases was defined as one or more immediate family members having a respiratory disease such as asthma, COPD, lung cancer, interstitial lung disease, or obstructive sleep apnea-hypopnea syndrome. The frequency of acute COPD exacerbations during the preceding year was defined as the new or worsening of at least two of the following respiratory symptoms: cough, phlegm, purulent sputum, wheezing, and dyspnea lasting over 48 h, (11) excluding left and right cardiac dysfunction, pulmonary embolism, pneumothorax, pleural effusion, and arrhythmia. Our team used self-reported distance to measure the distance from participants' homes and the location of outdoor activities to the nearest road. We asked participants to report the distance from their homes or where they performed outdoor activities to the main road with heavy traffic every day. The response options were <50 meters, 50–199 meters, and more than 200 meters (12, 13). Questionnaires were administered by trained investigators.

### Exposure assessment

Subjects were merged into a Geographical Information System (GIS). Geocoding (longitude and latitude) was done using home addresses. For the subject geocoding, we used cartographic data provided by the GIS Service of Guangzhou City: buildings, streets, and home addresses. We applied address geocoding techniques provided by Arcgis Pro3.0.2: a file extracted from the epidemiological questionnaire containing participants' addresses (street names and house numbers) was matched with vector data. Distances of houses from the main road were used to assess traffic-related pollution exposure. In Nanyuan Community, the main roads included Nanan Road and Zengcuo Road. In Hongqiao Community, the main roads included Xiaobei Road and Dongfengxi Road. In Xingang Community, the main roads included Xinjiaozhong Road and Dongxiaonan Road. Using GIS buffering and overlaying functionalities, we classified the population sample into three groups: <50 meters, 50–199 meters, and more than 200 meters, respectively (Figure 1).

### Spirometry

Spirometry testing was performed using a portable MasterScreen Pneumo Spirometer (CareFusion, CA, USA). The instrument calibration was checked before each assessment, following the methods and criteria recommended in the American Thoracic Society and European Respiratory Society guidelines (14). Spirometry tests were performed according to American Thoracic Society/European Respiratory Society standards (14) and were conducted by skilled physicians. Participants were excluded if they had allergies to medication or uncontrolled hypertension under the premise of albuterol sulfate (salbutamol) aerosol use. Prebronchodilator spirometry testing was done before the inhalation of 400 μg salbutamol, and postbronchodilator spirometry was conducted after 20 min following the same process. If the participant was using inhaled or oral respiratory medication to relieve airway obstruction or symptoms, we suggested they stop short-acting bronchodilator medications for 12 h or long-acting bronchodilator medications for 24 h before testing. We asked participants whether they used bronchodilator medications before performing spirometry; if they used medication, spirometry was performed the following day. We chose at least three acceptable curves and two repeatable measurement curves (maximum and submaximum values of FVC and FEV_1_ within 150 mL or 5%) when performing lung function tests.

The Ethics Committee of the First Affiliated Hospital of Guangzhou Medical University approved the study protocol (2013-37). All participants signed informed consent forms.

### Statistical analysis

Data were analyzed using IBM SPSS version 22.0 software (IBM Corp., Armonk, NY, USA). We used analysis of variance for continuous variables and the chi-squared test for categorical variables. Analysis of potential risk factors for COPD was performed using a logistic regression model in a stepwise estimation process, with risk factors as the dependent variable and age, sex, BMI, smoking status, family history of respiratory disease, and community district as a fixed covariate. Multivariate analysis of variance was used to evaluate lung function, with sex, smoking status, family history of respiratory disease, distance from residence, and location of outdoor activities to the nearest busy road as independent indices, age, and height as covariates, and lung function values as dependent variables. All *p*-values were double-sided, with a *p*-value of < 0.05 indicating statistical significance.

## Results

### Analysis of detection rate and risk factors in patients with COPD

**Figure 2:**
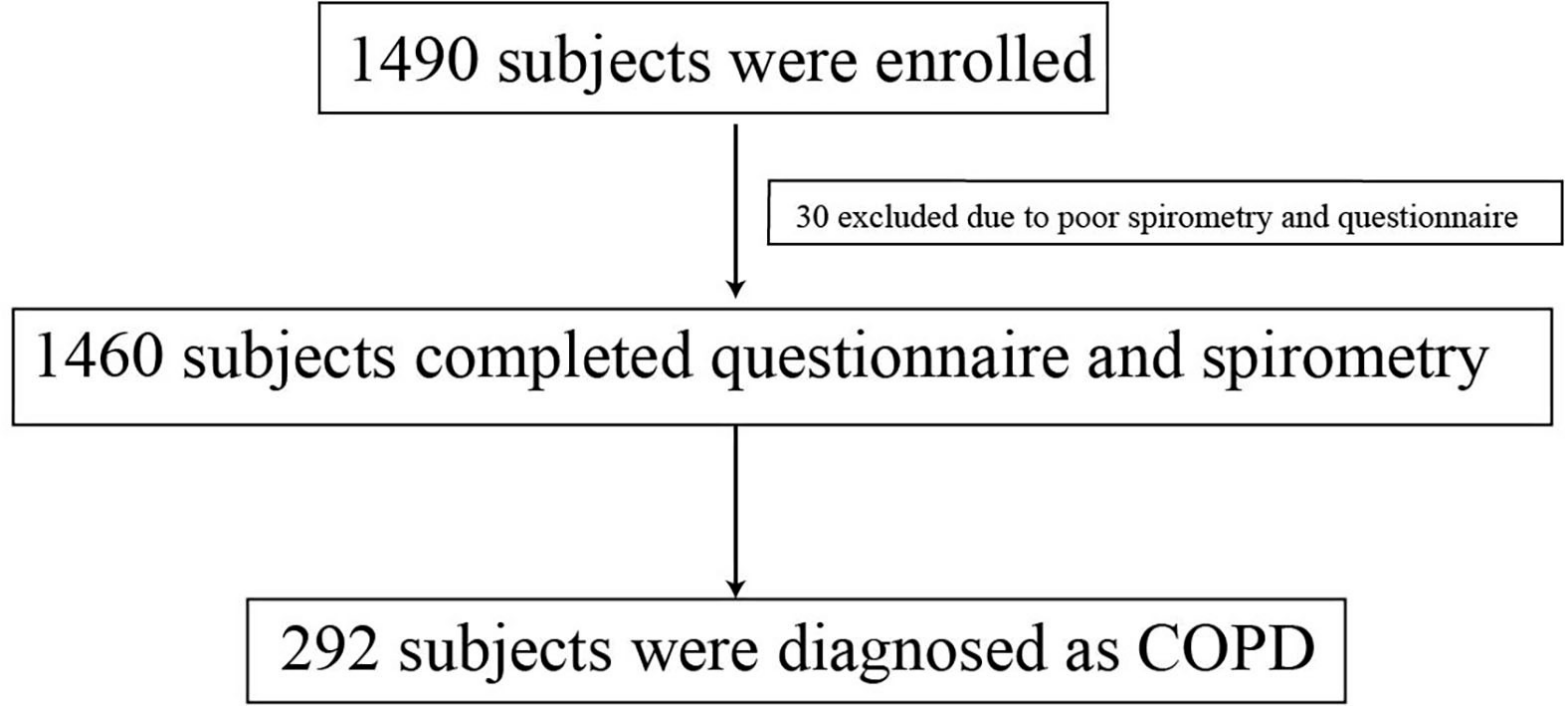
Flow chart. COPD, chronic obstructive pulmonary disease.

Of the 1,490 participants in this study, 1,460 completed the questionnaire and spirometry, and 292 individuals were diagnosed with COPD, with a prevalence of 20% (Figure 2). Of the 1,460 participants, 638 were men, among whom 38.1% had COPD; the adjusted odds ratio (OR) for the male sex was 3.47 (95% confidence interval [CI]: 2.10–5.74, *p* < 0.001), with the female sex as reference. With increasing age, the prevalence of COPD increased in the age groups 40–49 (1.4%), 40–59 (7.1%), 60–69 (21.7%), and ≥70 (33.3%) years, with the age group of 40–49 years as a reference; the adjusted ORs showed the same tendency (OR 2.83, 95% CI: 0.35–22.85, *p* = 0.329; OR 8.60, 95% CI: 1.11–66.69, *p* = 0.039; OR 20.14, 95% CI: 2.59–156.85, *p* = 0.004) in the age groups 40–59, 60–69, and ≥70 years, respectively (Table 1).

**Table 1:** Clinical characteristics and risk factors in patients with COPD.

	**N**	**COPD**		**OR (95% C*I*)**	***P*-values**	**Adjusted OR (95% C*I*)**	**Adjusted *P* values**
		***n***	**%**				
**Gender**					< 0.001		< 0.001
Male	638	243	38.1%	9.71 (6.98–13.50)		3.47 (2.10–5.74)	
Female	822	49	6.0%	1.00 (reference)		1.00 (reference)	
**Age, yrs**					< 0.001		< 0.001
40–49	72	1	1.4%	1.00 (reference)		1.00 (reference)	
40–59	396	28	7.1%	5.40 (0.72–40.35)	0.100	2.83 (0.35–22.85)	0.329
60–69	577	125	21.7%	19.64 (2.70–142.73)	0.003	8.60 (1.11–66.69)	0.039
≥70	415	138	33.3%	35.37 (4.86–257.28)	< 0.001	20.14 (2.59–156.85)	0.004
**Smoking status**					< 0.001		0.002
Never smokers	955	73	7.6%	1.00 (reference)		1.00 (reference)	
Former smokers	441	196	44.4%	9.67 (7.13-13.10)	< 0.001	2.84 (1.55-5.21)	0.001
current smokers	39	11	28.2%	4.75 (2.27-9.92)	< 0.001	1.54 (0.58-4.08)	0.388
**Smoking index, pack-years**					< 0.001		0.015
< 15	1,070	110	10.3%	1.00 (reference)		1.00 (reference)	
15–29.9	117	46	39.3%	5.65 (3.72–8.61)	< 0.001	1.36 (0.72–2.57)	0.336
30–44.9	112	56	50.0%	8.73 (5.74–13.28)	< 0.001	2.34 (1.24–4.42)	0.009
≥45	100	58	58.0%	12.05 (7.74–18.78)	< 0.001	2.42 (1.27–4.63)	0.007
**BMI, kg/m** ^2^					0.002		0.001
< 18.5	64	18	28.1%	2.76 (1.35–5.66)	0.006	2.73 (1.08–6.93)	0.034
18.5–24.9	752	171	22.7%	2.08 (1.26–3.42)	0.004	2.69 (1.46–4.97)	0.002
25–30	479	81	16.9%	1.44 (0.85–2.43)	0.178	1.52 (0.80–2.92)	0.203
≥30	161	20	12.4%	1.00 (reference)		1.00 (reference)	
**Whether the family has respiratory diseases**					< 0.001		< 0.001
No	1,045	179	17.1%	1.00 (reference)		1.00 (reference)	
Yes	406	112	27.6%	1.84 (1.41–2.42)		2.93 (2.03–4.23)	
**Distance from living place to closest busy roads**					0.016		0.367
< 50 m	383	79	20.6%	1.49 (1.07–2.09)	0.020	1.06 (0.70–1.61)	0.787
50–199 m	425	90	21.2%	1.54 (1.11–2.14)	0.009	1.33 (0.88–1.99)	0.175
≥200 m	580	86	14.8%	1.00 (reference)		1.00 (reference)	
**Outdoor activities away from the busy roads**					< 0.001		0.008
< 50 m	282	67	23.8%	1.96 (1.39–2.77)	< 0.001	1.54 (1.01–2.36)	0.047
50–199 m	355	87	24.5%	2.04 (1.48–2.82)	< 0.001	1.84 (1.23–2.76)	0.003
≥200 m	737	101	13.7%	1.00 (reference)		1.00 (reference)	

ORs were adjusted by confounding factors including age, sex, BMI, smoking status, family history of respiratory disease and community district. BMI, body mass index; OR, odds ratio; CI, confidence interval; COPD, chronic obstructive pulmonary disease.

The detection rates of COPD among never, former, and current smokers were 7.6, 44.4, and 28.2%, respectively. Compared with never smokers, the adjusted ORs of former and current smokers were 2.84 (95% CI: 1.55–5.21, *p* = 0.001) and 1.54 (95% CI: 0.54–4.08, *p* = 0.388), respectively. We also analyzed the smoking index, divided into four groups, as shown in Table 1. The detection rate of COPD increased with a higher smoking index, with rates of 10.3, 39.3, 50.0, and 58.0% in the groups 0–15, 15–29.9, 30–44.9, and more than 45 pack-years, respectively. With 0–15 pack-years as a reference, the adjusted OR ratios of the other three groups were 1.36 (95% CI: 0.72–2.57, *p* = 0.336), 2.34 (95% CI: 1.24–4.42, *p* = 0.009), and 2.42 (95% CI: 1.27–4.63, *p* = 0.007), respectively.

BMI has an important role in COPD, and we found that the lower the BMI, the higher the incidence of COPD, with 28.1, 22.7, 16.9, and 12.4% in participants with BMIs 0–18.5, 18.5–24.9, 25–30, and >30 kg/m^2^, respectively. With BMI >30 kg/m^2^ as a reference, the adjusted ORs for BMI 0–18.5, 18.5–24.9, and 25–30 kg/m^2^ were 2.73 (95% CI: 1.08–6.93, *p* = 0.034), 2.69 (95% CI: 1.46–4.97 *p* = 0.002), and 1.52 (95% CI: 0.80–2.92, *p* = 0.203), respectively.

Family history of respiratory disease is a risk factor for COPD. Participants with a family member with a respiratory disease, such as asthma or chronic bronchitis, had a higher prevalence of COPD than their counterparts with such a family history (27.6 vs. 17.1%, *p* < 0.001). The adjusted OR for individuals with a family history of respiratory diseases was 2.93 (95% CI: 2.03–4.23, *p* < 0.001).

We divided the distance from the participant's residence to the nearest busy road into three groups. The incidence rates of COPD by distance were 20.6, 21.2, and 14.8% among participants who lived <50 meters, 50–199 meters, and ≥200 meters from a busy road, respectively. Adjusted ORs were 1.06 (95% CI: 0.70–1.61, *p* = 0.787) and 1.33 (95% CI: 0.88–1.99, *p* = 0.175) in the groups <50 meters and 50–199 meters, respectively, with ≥200 meters as the reference. Similarly, in the analysis regarding the distance of outdoor activities from busy roads, the incidence rates of COPD were 23.8, 24.5, and 13.7%, with adjusted ORs of 1.54 (95% CI: 1.01–2.36, *p* = 0.047) and 1.84 (95% CI: 1.23–2.76, *p* = 0.003) for <50 meters and 50–199 meters, respectively, with ≥200 meters as reference. All OR values were statistically significant (*p* < 0.05).

To sum up, the closer the participants' residence or the location of outdoor activities is to a busy road, the higher the detection rate of COPD. Male sex, age 40–80 years, a lower BMI, smoking, and a family history of respiratory disease were risk factors for COPD.

### Differences in pulmonary function according to high-risk factors

After adjusting for high-risk factors, pulmonary function values were significantly different among groups before and after bronchodilation tests. Although lung function tests are affected by various factors (sex, age, smoking status, and BMI), risk factors were used statistically as independent indexes, with age and height as covariates and lung function indicators as dependent variables, such that multivariate analysis of variance was used.

The study findings showed that the distance from participants' residence and outdoor activities to a busy road, male sex, smoking history, and family history of respiratory disease were important factors that resulted in changes in the values of FEV_1_ and FVC (Table 2). The post-FEV_1_ values (2.15 ± 0.3 L vs. 2.07 ± 0.54 L vs. 2.06 ± 0.57 L, *p* = 0.030) and post-FVC values (2.82 ± 0.68 L vs. 2.76 ± 0.70 L vs. 2.65 ± 0.63 L, *p* < 0.001) were higher for residence <50 meters, compared to 50–199 meters, and ≥200 meters from a busy road. Pre-FEV_1_/FVC values were 74.3 ± 10.0% vs. 73.17 ± 11.93% vs. 75.43 ± 9.69%, *p* = 0.003 and post-FEV_1_/FVC values were 76.82 ± 10.47% vs. 75.76 ± 12.14% vs. 78.41 ± 9.65%, *p* = 0.001 for residence <50 meters, 50–199 meters, and ≥200 meters from a busy road. The further participants engaged in outdoor activities from a busy road, the lower the values of post-FVC (2.85 ± 0.72 L vs. 2.77 ± 0.69 L vs. 2.67 ± 0.64 L, *p* = 0.001) for <50 meters, 50–199 meters, and ≥200 meters. Pre-FEV_1_/FVC and post-FEV_1_/FVC values were higher with a further distance of outdoor activities from the main road. As shown in Table 2, female participants had higher pre-FEV_1_/FVC values (78.1 ± 7.0% vs. 68.7 ± 12.8%, *p* < 0.001) and post-FEV_1_/FVC values (81.2 ± 6.8% vs. 70.9 ± 12.9%, p < 0.001) than their male counterparts. Individuals with a family history of respiratory disease had lower pre-FEV_1_/FVC (72.4 ± 12.2% vs. 74.6 ± 10.5%, *p* = 0.001) and post-FEV_1_/FVC (75.2 ± 12.6% vs. 77.2 ± 10.6%, *p* = 0.003) values than their counterparts without such a family history.

**Table 2:** Multivariate analysis of variance in pulmonary function.

	**Pre-FVC¶ (*n =* 1,457, L)**	**Pre-FEV1¶ (*n =* 1,457, L)**	**Pre-FEV1/FVC¶ (*n =* 1,457, %)**	**Post-FVC♮ (*n =* 1,420, L)**	**Post-FEV1♮ (*n =* 1,420, L)**	**Post-FEV1/FVC♮ (*n =* 1,420, %)**
**Dwelling away from busy roads**						
<50 m	2.81 ± 0.68	2.09 ± 0.55	74.3 ± 10.0	2.82 ± 0.68	2.15 ± 0.3	76.82 ± 10.47
50–199 m	2.74 ± 0.70	1.99 ± 0.56	73.17 ± 11.93	2.76 ± 0.70	2.07 ± 0.54	75.76 ± 12.14
≥200 m	2.65 ± 0.66	2.00 ± 0.52	75.43 ± 9.69	2.65 ± 0.63	2.06 ± 0.57	78.41 ± 9.65
*P* value	0.002	0.014	0.003	<0.001	0.030	0.001
**Outdoor activities away from the busy roads**						
<50 m	2.84 ± 0.73	2.06 ± 0.57	73.16 ± 11.03	2.85 ± 0.72	2.13 ± 0.55	75.58 ± 11.27
50–199 m	2.73 ± 0.69	1.98 ± 0.56	72.92 ± 11.67	2.77 ± 0.69	2.05 ± 0.54	75.34 ± 12.10
≥200 m	2.67 ± 0.65	2.02 ± 0.52	75.60 ± 9.67	2.67 ± 0.64	2.08 ± 0.52	78.52 ± 9.66
*P* value	0.007	0.018^♮^	<0.001	0.001	0.022^♮^	<0.001
**Gender**						
Male	3.10 ± 0.70	2.14 ± 0.65	68.7 ± 12.8	3.15 ± 0.67	2.22 ± 0.63	70.9 ± 12.9
Female	2.43 ± 0.51	1.9 ± 0.42	78.1 ± 7.0	2.41 ± 0.47	1.95 ± 0.41	81.2 ± 6.8
*P* value	<0.001	<0.001	<0.001	<0.00	<0.001	<0.001
**Smoke**						
No	3.17 ± 0.73	2.17 ± 0.62	68.1 ± 11.4	3.23 ± 0.70	2.25 ± 0.61	70.0 ± 11.6
Yes	2.62 ± 0.63	1.97 ± 0.52	75.4 ± 10.5	2.62 ± 0.61	2.03 ± 0.51	78.3 ± 10.5
*P* value	<0.001	<0.001	<0.001	<0.001	<0.001	<0.001
**Family members have respiratory diseases**						
No	2.72 ± 0.69	2.02 ± 0.55	74.6 ± 10.5	2.73 ± 0.68	2.09 ± 0.53	77.2 ± 10.6
Yes	2.72 ± 0.66	1.96 ± 0.55	72.4 ± 12.2	2.74 ± 0.67	2.03 ± 0.53	75.2 ± 12.6
*P* value	0.923	0.079	0.001	0.762	0.09	0.003

¶, ♮ Best value before and after bronchodilator, respectively. ^#^ Multivariate analysis of variance was evaluated using sex, smoking status, family history of respiratory disease, and distance from residence or outdoor activities to closest busy roads as independent indexes and with age and height as covariates. FVC, forced vital capacity; FEV_1_, forced expiratory volume in 1 second.

## Discussion

Our study findings indicated that respiratory health risks exist for people living and engaging in outdoor activities close to the main road. Our study used participants' residences and outdoor activities as a proxy for traffic-related air pollution exposure. Thus, participants living or participating in outdoor activities at the same distance from a busy road were estimated to have the same exposure levels. Owing to various factors related to the development of COPD, a logistic regression model was fitted, adjusting for age, sex, BMI, smoking status, smoking index, family history of respiratory disease, and distance from the home and outdoor activities to the closest busy road. We found that the distance of outdoor activities from the main road was an independent risk factor for developing COPD, with an adverse influence on lung function.

In our study, we recruited community residents who lived in central Guangzhou and were aged 40–80 years. The detection rate of COPD was 20%, higher than that in a study by Wang et al. (15), who estimated that the prevalence of COPD was 13.7% in the same age group during 2012–2015. The difference in the findings may be due to different regions and insufficient samples. We found that male sex, older individuals with a lower BMI, a smoking history, and a family history of respiratory diseases (chronic bronchitis, emphysema, asthma, and COPD among immediate family members) were more likely to have COPD. Our research showed a higher risk of COPD among ex-smokers than current smokers, which is similar to a large population-based survey that also found a higher prevalence and ORs for COPD among ex-smokers than current smokers (16). This could be related to lifetime exposure to smoking and the longer lifespan of ex-smokers. Smokers frequently lack information about smoking cessation and the health risks associated with smoking, and as a result, they do not quit smoking unless they develop severe diseases (16).

After adjusting for sex, age, BMI, smoking status, and family history of respiratory disease, the distance of outdoor activities from busy roads was an independent risk factor for COPD. The distance from the participant's residence to a busy road was not a significant risk factor for COPD development after adjusting the factors above. However, we found that the closer the residence and outdoor activities were to busy roads, the higher the COPD detection rates and ORs. Living near dense traffic could increase exposure to traffic-related air pollution, including combustion products, nitrogen dioxide, and ultrafine PM (17, 18). In the Canadian Census Health and Environment Cohort (CanCHEC) study during 1991–2001, increased exposure to local roads and living near a major highway were linked to increased mortality owing to respiratory diseases, even after adjusting for air pollution concentrations (19). However, the odds ratio of developing COPD for living <50 meters from a busy road was lower than that for living at a 50–199-meter distance in our research. This may be because residents in the <50 meters group used more ways to decrease the influence of air pollution, such as by closing windows. People living further from the main road might not implement such protective measures, resulting in greater exposure to air pollution. Other possible causes for our findings must be further explored. Nuvolone et al. found that the highest values of lung function were observed in the intermediate group (100–250 meters) than in the 100-meter and 250–800-meter groups, which suggests that respiratory health impairment owing to traffic-related air pollution occurs at a distance over 100 meters (20) vs. other studies showing negative effects of living near a busy road up to a distance of 500 meters (9).

The World Health Organization estimates that 22% of global mortality and disability can be attributed to environmental causes. Air pollution contributes to approximately 9% of COPD cases (21). Traffic-related air pollution mainly comes from motor vehicle exhaust and road dust (22). As a megacity in China, Guangzhou currently has more than 2 million cars. With the increasing number of vehicles, the traffic volume on main roads in urban areas is large, and congestion during peak hours is serious. As vehicle emissions increase, the concentration of incomplete combustion materials also increases (23). Research on the mechanism of air pollutants such as PM, nitrogen dioxide, and ozone in the development and exacerbation of asthma showed that air pollutants are associated with the induction of both eosinophilic and neutrophilic inflammation driven by stimulation of the airway epithelium and increased pro-inflammatory cytokine production, oxidative stress, and DNA methylation changes (24). Higher ambient air pollution and exposure to nitrogen oxides and PM can result in respiratory mortality and a decline in lung function (25), which can lead to the development of COPD. In the 1970s, Fletcher found that the decline of lung function, especially FEV_1_, could be sustained over a lifetime. In vulnerable people, smoking leads to a risk of irreversible obstructive changes because tobacco smoke may cause exposure to PM (26). Schikwski et al. found that chronic exposure to air pollution and living close to a major road increased the risk of developing COPD and had a harmful effect on lung function in women (27). Téllez-Rojo et al. found that an increase of 10 μg/m^3^ in PM_10_ was related to a 4.1% increase in COPD deaths (28). A review found that every 5-μg/m^3^ increment in PM_2.5_ was associated with a decrease of 1.18% for FVC, 1.46% for FEV_1_, 1.65% for maximum mid-expiratory flow (MMEF), and 0.21% for FEV_1_/FVC; the interpretation of the findings was that long-term exposure to PM_2.5_ would result in faster lung function decline, similar to the findings of a longitudinal cohort study conducted in Taiwan (29). Kwon et al. reported that long-term exposure to PM_10_ was related to both spirometry values and imaging phenotypes in COPD. With an annual 4.4-μg/m3 exposure to PM_10_, there was an interquartile range difference of 0.13 L lower FVC (30). It is difficult to calculate the amount of traffic-related air pollution exposure during exercise owing to the lack of a complex model to assess multiple and variable exposures to air pollutants and pollutant concentrations (31). We found that the closer to busy road participants engaged in outdoor activities, the poorer their lung function. For example, lung function values of FEV_1_/FVC were lower, and the incidence rate of COPD was higher with outdoor activities carried out closer to the main road, suggesting that lung function decline may be related to air pollution from traffic. In a large cohort study in the United Kingdom, the effect of declining lung function was significant even at very low levels of ambient PM2.5. Air pollution had a larger impact on lung function decline in male individuals, those from low-income households, those with occupations in which they were exposed to air pollution, and individuals with obesity (25); these findings were similar to our study's findings. However, unlike other research (20), we did not adjust for occupational exposure as a confounding factor because there was no significant difference in occupational exposure before adjusting for confounders (data not shown).

There are several strengths in our study. This observational study enrolled a large sample in the Guangdong district. This was the first study to use distance from a busy road as an indicator of traffic-related air pollution as an important risk factor for COPD in communities in Guangzhou. In this study, the extent of individual air pollution exposure was measured using the distance of participants' residences and the location of outdoor activities from busy roads. We described the relationship between traffic-related air pollution and COPD in some urban areas of Guangdong Province. Additionally, the findings of this study were consistent with those of other studies in that we identified male sex, smoking, older age, low BMI, and a family history of respiratory disease as risk factors for developing COPD (32).

Despite the strengths, this study also has some limitations. First, some questionnaires had missing responses, which could introduce bias. Second, only urban residents participated in this study; more participants who lived in rural areas were finally enrolled. Third, we only queried the distance from participants' residences and the location of outdoor activities to a busy road; we did not measure the precise distance. Additionally, we did not calculate or evaluate the composition or concentration of air pollutants because we could not obtain real-time air pollution data. Finally, we only used cross-sectional data in this study. Further follow-up studies are expected to analyze the change in lung function among different groups based on the distance of their residence or outdoor activities from main roads.

## Conclusion

The distance of outdoor activities from main roads was a risk factor in the development of COPD and had a negative effect on lung function, suggesting that traffic-related environmental pollution is associated with respiratory health. It is important to take steps to reduce vehicle exhaust emissions and traffic-related air pollution to enhance people's health.

## Data availability statement

The raw data supporting the conclusions of this article will be made available by the authors, without undue reservation.

## Ethics statement

The studies involving human participants were reviewed and approved by the Ethics Committee of the First Affiliated Hospital of Guangzhou Medical University approved this study protocol (2013-37). All subjects signed informed consent. The patients/participants provided their written informed consent to participate in this study.

## Author contributions

PR, YZ, BL, JZ, SL, HP, and JP designed the project and planned the statistical analysis. JZ, SL, JP, and ZW drafted and revised the paper. JZ, SL, HP, ZD, CL, NL, LT, JX, and JL collected and monitored data collection. All authors approved the final draft of the manuscript for publication.
